# Neutralization Assessments Reveal High Cardiothoracic Ratio and Old Age as Independent Predictors of Low Neutralizing Antibody Titers in Hemodialysis Patients Receiving a Single Dose of COVID-19 Vaccine

**DOI:** 10.3390/jpm12010068

**Published:** 2022-01-07

**Authors:** Chun-Yu Chen, Kuan-Ting Liu, Shin-Ru Shih, Jung-Jr Ye, Yih-Ting Chen, Heng-Chih Pan, Heng-Jung Hsu, Chiao-Yin Sun, Chin-Chan Lee, Chun-Ying Wu, Chi-Chun Lai, I-Wen Wu

**Affiliations:** 1 Department of Nephrology, Chang Gung Memorial Hospital, Keelung 204, Taiwan; shone@cgmh.org.tw (C.-Y.C.); b9402031@cgmh.org.tw (Y.-T.C.); balour@cgmh.org.tw (H.-C.P.); r5267@cgmh.org.tw (H.-J.H.); sun3970@cgmh.org.tw (C.-Y.S.); leefang@cgmh.org.tw (C.-C.L.); 2 College of Medicine, Chang Gung University, Taoyuan 333, Taiwan; ccl404@cgmh.org.tw; 3 Research Center for Emerging Viral Infections, College of Medicine, Chang Gung University, Taoyuan 333, Taiwan; jeff31602@gmail.com (K.-T.L.); srshih@mail.cgu.edu.tw (S.-R.S.); 4 Graduate Institute of Biomedical Science, College of Medicine, Chang Gung University, Taoyuan 333, Taiwan; 5 Department of Medical Biotechnology and Laboratory Science, College of Medicine, Chang Gung University, Taoyuan 333, Taiwan; 6 Graduate Institute of Medical Biotechnology and Laboratory Science, College of Medicine, Chang Gung University, Taoyuan 333, Taiwan; 7 Department of Infectious Diseases, Chang Gung Memorial Hospital, Keelung 204, Taiwan; loyawise@cgmh.org.tw; 8 Department of Laboratory Medicine, Chang Gung Memorial Hospital, Keelung 204, Taiwan; hla0861@cgmh.org.tw; 9 Department of Ophthalmology, Chang Gung Memorial Hospital, Keelung 204, Taiwan

**Keywords:** COVID-19 vaccine, neutralizing antibodies, clinical response, hemodialysis, effectiveness

## Abstract

Background: Data are lacking regarding predictors of quantification of neutralizing antibodies (nAbs) based on severe acute respiratory syndrome coronavirus 2 (SARS-CoV-2) 50% neutralization titer (NT_50_) after a single dose of COVID-19 vaccine in hemodialysis (HD) patients. Methods: This prospective single-center study enrolled 200 HD patients and 82 healthy subjects to estimate antibodies against the SARS-CoV-2 viral spike protein 1 and receptor-binding domain after a first dose of a COVID-19 vaccine (ChAdOx1 or mRNA-1273), measured by enzyme-linked immunosorbent assay and applied spline-based generalized additive model regression analysis to predict NT_50_ converted to international units. Results: After the first dose of ChAdOx1, multiple linear regression showed that age (*p =* 0.011) and cardiothoracic ratio (*p* = 0.002) were negatively associated with NT_50_. Older age (OR = 0.958, *p =* 0.052) and higher cardiothoracic ratio (OR < 0.001, *p =* 0.037) could predict negative humoral response (NT_50_ < 35.13 IU/mL). NT_50_ was lower in HD patients compared with healthy controls receiving ChAdOx1 (10.68 vs. 43.01 IU/m, *p* < 0.001) or mRNA-1273 (36.39 vs. 262.2 IU/mL, *p* < 0.001). ChAdOx1 elicited lower GMTs than mRNA-1273 in the HD cohort (10.68 vs. 36.39 IU/mL, *p* < 0.001) and in healthy controls (43.01 vs. 262.22 IU/mL, *p* < 0.001). Conclusion: High cardiothoracic ratio and old age could independently predict a decline in nAb titers in an HD cohort vaccinated with a single dose of ChAdOx1.

## 1. Introduction

The novel coronavirus, severe acute respiratory syndrome coronavirus 2 (SARS-CoV-2), has been declared a global emergency, affecting 263 million people worldwide, and continues to engender a tremendous global burden [1]. Advanced age, diabetes mellitus, cancer, cardiovascular disease, chronic liver disease, hypertension, and smoking have been identified as risk factors linked to the incidence of coronavirus disease 2019 (COVID-19) [2,3,4]. Higher risks of mortality caused by COVID-19 are found in patients with older age, more comorbidities, and immune dysfunction [3,5].

Once patients have reached stage 5 of chronic kidney disease (estimated glomerular filtration rate < 15 mL/min/1.73 m^2^), in combination with signs and symptoms owing to uremia—including uremic bleeding, protein–energy wasting, fluid overload, pericarditis, refractory metabolic acidosis, or hyperkalemia—renal replacement therapy (RRT) should be administered. Hemodialysis (HD) is the most prevalent method of RRT in Taiwan [6]; however, common acute complications regarding HD deserve our attention, such as intradialytic hypotension, cramps, chest pain, and vascular access infection [7]. Acute kidney injury requiring RRT frequently occurs in severe COVID-19 [8]. Furthermore, maintenance HD patients are also more vulnerable to COVID-19, because of greater age, coexistence of comorbidities, and immune-dysregulated status on account of uremia, comorbidities, dialysis-procedure-related biocompatibility, and microinflammation [9,10].

In-center HD patients are an extremely vulnerable group; they are immunocompromised, require lifelong attendance at crowded dialysis facilities, and often have substantially attenuated vaccine response. The incidence rate of COVID-19 was approximately 7.7%—and the overall mortality rate could have been as high as 22.4%—in the HD population during the first wave of the global pandemic [11]. Therefore, proactive infection-preventive measures should be adopted, and extensive vaccination is needed in this fragile population.

COVID-19 vaccination has been proven to reduce the incidence rate, lower the morbidity and mortality risk, and remain effective even in patients with immunocompromised status. Although the immunogenicity of COVID-19 vaccines seems to be attenuated in immunocompromised subjects in comparison to the general population, the potential for severe COVID-19 in this population offsets the uncertainties [12,13,14]. The cellular response and humoral response in HD patients were reported to be inferior to those of the general population. Neutralizing antibody (nAb) responses to the adenoviral vector vaccine ChAdOx1 (Oxford–AstraZeneca) were tempered in HD patients, and one-third of patients receiving the attenuated adenovirus Ad26.COV2.S vaccine (Johnson & Johnson) failed to seroconvert even 28–60 days post-vaccination [15,16]. The phenomenon of reduced humoral response was also found in uremic patients and kidney allograft recipients who were vaccinated with a first dose of the mRNA vaccine BNT162b2 (Pfizer/BioNTech) [17,18].

Taiwan has attained efficacious containment of SARS-CoV-2 transmission, keeping up to 253 days of zero domestic confirmed cases since the start of the devastating worldwide pandemic in 2020. However, a second wave of the COVID-19 pandemic has emerged in northern Taiwan since 14 May 2021, with multiple clusters of transmission, and has spread into other regions of the country, leading to 753 deaths within two months [19]. The overall coverage rate of the first dose was only 1.5% during the time of community outbreak, when vaccine supply was extremely lacking. The process of national general vaccination lags behind most western countries undergoing more severe epidemics. The first phase of Taiwan’s vaccination program mainly used imported ChAdOx1 (Oxford–AstraZeneca), and the national vaccination policy has prioritized inoculation in the elderly, severely ill, and immunocompromised populations. Because of several clustered infection events in HD facilities in northern Taiwan, contributing to grave mortalities, HD patients were listed as priority targets for vaccination in the middle of June 2021 [20]. Vaccination was also vigorously suggested by the International Society of Nephrology, the American Society of Nephrology, the European Dialysis and Transplant Association, and the Taiwan Society of Nephology [21,22,23,24,25].

As Taiwan has the highest prevalence of end-stage renal disease (ESRD) worldwide, effective vaccination to prevent COVID-19 infection in this vulnerable population remains crucial. However, the effectiveness of vaccination in ESRD patients, and how their immune responses differ from those of the general population, is not completely understood. For the tempered post-vaccination humoral response in uremic patients, an accurate and safe method to rapidly quantify the nAbs and to identify the most vulnerable HD patients remains crucial; those who have low titers of nAbs might be prioritized for booster shots. To fill this knowledge gap, we conducted an observational cohort study to compare the titers of nAbs between ESRD patients and subjects with normal renal function who received a single dose of a COVID-19 vaccine. We also delineated possible factors associated with responses to vaccination. This information may provide the necessary insight to design an effective vaccination program for ESRD patients [26].

## 2. Materials and Methods

### 2.1. Study Design and Patient Characteristics

**Figure 1:**
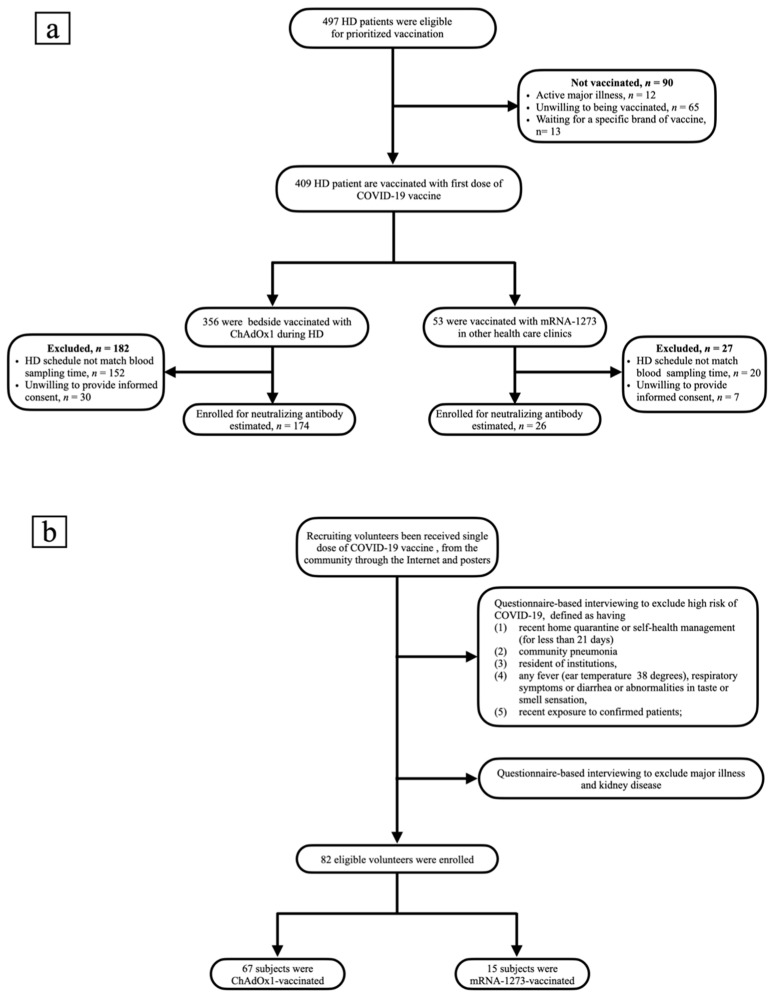
(**a**) Flow chart of patients on hemodialysis selected for neutralizing antibody measurement. (**b**) Flow chart of recruitment of healthy controls.

This was an observational, prospective, single-center study to evaluate nAb response in HD patients after a single dose of COVID-19 vaccination carried out in Chang Gung Memorial Hospital Keelung Branch in Taiwan. Bedside COVID-19 inoculation policy for HD patients was promulgated by the national health authorities and the Taiwan Society of Nephrology, who uniformly provide the ChAdOx1 vaccine to HD facilities for injection. Patients older than 18 years and who had been on HD for >3 months were screened. Those patients with active malignancy, under immunosuppressive treatment, with a Child–Pugh liver cirrhosis score worse than A, with a history of prior infection with SARS-CoV-2, who had previously been vaccinated at another healthcare center, or who were unwilling to be vaccinated were excluded from nAb titer analysis. The same enrollment criteria were used for the healthy control group (normal renal function subjects) recruited from the community. Overall, 497 patients were receiving long-term maintenance HD in our hospital-affiliated dialysis center, and 356 of them received bedside ChAdOx1 vaccination, while 53 patients were vaccinated with mRNA-1273. A total of 200 vaccinated HD patients were enrolled into the study, comprising 174 patients receiving the ChAdOx1 vaccine and 26 patients receiving the mRNA-1273 vaccine (Figure 1a). The healthy control group comprised 82 individuals, including 67 ChAdOx1 subjects and 15 mRNA-1273 subjects (Figure 1b). Furthermore, SARS-CoV-2 rapid antigen tests were mandatory in all of our HD patients on a weekly basis, possibly helping to avoid potential false elevation of humoral response due to infection. Since national health policy recommends a 12-week interval before the second dose of vaccination, none of our patients have received the subsequent dose. Differences in nAb titers between HD patients and healthy controls, as well as between the two types of vaccine, were compared. In addition, clinical, laboratory, and dialysis-related parameters were recorded and correlated with serum titers of nAbs.

This study was performed in accordance with the Declaration of Helsinki, and was approved by the Ethics Committee of the Institutional Review Board at Chang Gung Memorial Hospital (IRB: 202001041A3C604 and 202100854B0A3). Written informed consent was obtained from all study participants.

### 2.2. Sample Collection

Blood samples were collected after overnight fasting, and were delivered immediately (within 4 h of collection) to the laboratory for biochemical analyses, complete blood counts, and antibody titers. Blood samples of HD patients were collected during a single HD session via venous chamber before dialysis treatment. A fraction of the samples were transferred to chilled tubes and centrifuged at 3000× *g* for 10 min at 4 °C to obtain the sera. Lipemic or hemolyzed sera were discarded.

### 2.3. Immunogenicity Response Assessment

Immunogenicity was assessed by measuring nAb response on day 56 in HD patients and after a median of 30 (26–50) days in healthy control subjects after administering the first dose of COVID-19 vaccine, using the Formosa Biomedical Technology MeDiPro SARS-CoV-2 Antibody ELISA assay, which detects antibodies against the SARS-CoV-2 viral spike protein 1 (S1) and receptor-binding domain (RBD). MeDiPro is a kit for quantifying nAbs using technology transferred from the Research Center for Emerging Viral Infections, Chang Gung University, and has been approved by the Taiwan Food and Drug Administration (No. 1106803303); the data for S1 and RBD fusion proteins can accurately predict the SARS-CoV-2 50% neutralization titer (NT_50_) under a two-variable generalized additive model and WHO international unit conversion (Supplementary Materials). According to the manufacturer, this test has 92.2% (95% CI, 84.0–96.4%) sensitivity and 93% (95% CI, 81.4–97.6%) specificity.

### 2.4. Quantifying nAbs by a Two-Variable Generalized Additive Model

The MeDiPro SARS-CoV-2 Antibody ELISA kit was designed to detect SARS-CoV-2 nAbs in the serum, based on the binding affinity of S1 and RBD to antibodies. The RBD is the major binding site of nAbs. S1 covers the RBD and several other regions, which are also imperative for nAb binding. The assay combines each of the S1 and RBD ELISA unit (EU) values, and applies spline-based generalized additive model (GAM) regression analysis (using S1 and RBD as two predictors) to predict NT_50_ by combining multiple smooth functions.

### 2.5. WHO International Standard Unit (IU) Conversion

In order to facilitate the conversion of geometric mean titers (GMTs) of NT_50_ to international units, WHO international standard (IS) sera (20/130, 20/136, and 20/268) were obtained from the National Institute for Biological Standards and Control (NIBSC). IS sera were used to obtain nAb titers in IU/mL. The NT_50_ values for WHO IS sera were determined by a live virus microneutralization assay. Each standard serum sample was tested in duplicate, except for 20/130. To convert NT_50_ to IU/mL, a neutralizing assay was developed to calculate the GMTs of the NIBSC serum samples. Values of <12.31 IU/mL (NT_50_ < 2.56) were defined as negative humoral response, values between 12.31 and 35.13 IU/mL (2.56 ≤ NT_50_ < 8) were defined as weakly positive humoral response, and values > 35.13 IU/mL (NT_50_ > 8) were defined as positive humoral response. For the cutoff values of NT_50_ (12.31 IU/mL), we used the ELISA’s limitation of detection (LOD). When we enter the value of the LOD into the model, we get the value of 12.31 IU/mL. The cutoff value of 35.13 IU/mL comes from converting the lowest neutralizing titer (NT) to IU/mL. In our clinical practice, the serial dilutions of the virus neutralization assay started from 1:8, because any lower dilution of serum is toxic to the cells, and causes bias in determining the titer. As we input NT = 8 to the model, we get the value of 35.13 IU/mL.

### 2.6. Statistical Analysis

The demographics and clinical characteristics of 3 groups with different NT_50_ after a first dose of ChAdOx1 vaccination were compared by one-way analysis of variance (ANOVA) in order to compare the normally distributed variables, with values expressed as means with standard deviations, while categorical variables were tested using the chi-squared test, and the nonparametric independent Kruskal–Wallis test was performed to compare the non-normally distributed variables, with values expressed as medians with interquartile ranges.

Simple linear regression and multiple regression were both applied to examine the association between independent variables and NT_50_ after the first dose of ChAdOx1 vaccination. The non-normally distributed variables were logarithmically transformed, and then were subjected to regression analysis. Model 1 multiple regression included all variables, while Model 2 multiple regression excluded anti-S1 and anti-RBD antibodies with age adjustment.

To assess the associations between positive humoral response and clinically important dialysis-related variables after the first dose of ChAdOx1 vaccination, the odds ratio to produce an adequate amount of nAbs (predicted NT_50_ > 35.13 IU/mL) was calculated by binary univariate and multivariable logistic regression, using the Enter method. 

To compare the nAb responses between the two kinds of vaccine, age was compared using Student’s *t*-test, gender and humoral response were compared using the chi-squared test, and non-normally distributed data of predicted NT_50_ and GMTs were compared by nonparametric independent Mann–Whitney U test, with values expressed as medians with interquartile ranges.

The receiver operating characteristic (ROC) curves were plotted to predict the probability of a binary outcome, including age vs. positive humoral response and cardiothoracic ratio vs. positive humoral response. Differences were examined using the area under the receiver operating characteristic (AUROC) curve.

Continuous variables were tested for normal distribution using skewness, kurtosis, and the Kolmogorov–Smirnov test. All statistical analyses were two-tailed, and a value of *p* < 0.05 was considered statistically significant. Data were analyzed using the Statistical Package for the Social Sciences (SPSS, Inc., Chicago, IL, USA) version 26.0 for Mac. GraphPad Prism version 8 (GraphPad Software, Inc., San Diego, CA, USA) was used to calculate GMTs with 95% confidence intervals (CIs), and to generate all of the graphs.

## 3. Results

### 3.1. Study Design and Subject Characteristics

A total of 200 vaccinated HD patients were enrolled, comprising 174 patients receiving the ChAdOx1 vaccine and 26 patients receiving the mRNA-1273 vaccine (Figure 1a). The healthy control group enrolled 82 individuals, including 67 ChAdOx1 subjects and 15 mRNA-1273 subjects (Figure 1b). The enrolled HD subjects had comparable age, albumin, Kt/V, and cardiothoracic ratio to non-enrolled HD subjects (Table S1). The mRNA-vaccinated HD patients had a higher percentage of liver cirrhosis (*p =* 0.002) and a lower percentage of cardiovascular disease (*p =* 0.011) than ChAdOx1-vaccinated HD patients (Table S2). The Pearson’s correlation coefficients (r) between age and NT_50_ were −0.3892 (*p* < 0.0001) in ChAdOx1-vaccinated subjects and −0.3889 (*p =* 0.012) in mRNA-1273-vaccinated subjects (Figure S1). Among the HD cohort, ChAdOx1-vaccinated patients had lower amounts of anti-S1 (2.28 vs. 3.89 EU/mL, *p =* 0.001) and anti-RBD (2.14 vs. 3.16 EU/mL, *p* < 0.001) antibodies than mRNA-1273-vaccinated patients. HD patients vaccinated with either ChAdOx1 or mRNA-1273 had similar age (64.97 ± 13.20 vs. 68.51 ± 10.35, *p =* 0.192), percentage of diabetes (55.2% vs. 57.7%, *p =* 0.809), albumin (4.03 ± 0.37 vs. 4.10 ± 0.40, *p =* 0.388), Kt/V (1.64 ± 0.34 vs. 1.76 ± 0.26, *p =* 0.079), and cardiothoracic ratio (0.52 ± 0.07 vs. 0.52 ± 0.07, *p =* 0.928) (Table S2). 

### 3.2. NT_50_ and Clinical Characteristics of ChAdOx1-Vaccinated HD Patients

HD patients who received a first dose of ChAdOx1 vaccination were stratified according to NT_50_ of nAbs. The high-titer patients were more likely to have higher serum ALT and lower cardiothoracic ratio (Table 1). The baseline comorbidities and medications were similar among the three NT_50_ groups. Simple linear regression analysis for factors associated with NT_50_ showed that anti-S1 Abs and anti-RBD antibodies (β ± SE: 1.841 ± 0.065, *p* < 0.001; and β ± SE: 2.250 ± 0.0106, *p* < 0.001, respectively) were positively associated with NT_50_, while cardiothoracic ratio (β ± SE: −2.026 ± 0.729, *p =* 0.006) was negatively associated with NT_50_ (Table 2). Moreover, we performed multiple linear regression analysis with a backward stepwise selection method to estimate factors associated with NT_50_. After adjusting all variables (Model 1), age (β ± SE: −0.003 ± 0.001, *p =* 0.046), hemoglobin (β ± SE: −0.136 ± 0.056, *p =* 0.017), uric acid (β ± SE: −0.021 ± 0.010, *p =* 0.038), ferritin (β ± SE: −0.119 ± 0.048, *p =* 0.016), and Ca × P product (β ± SE: −0.003 ± 0.001, *p =* 0.006) were significantly, independently, and negatively associated with NT_50_, while anti-Si antibodies (β ± SE: 1.490 ± 0.128, *p <* 0.001), anti-RBD antibodies (β ± SE: 0.589 ± 0.165, *p =* 0.001), red blood cells (β ± SE: 2.787 ± 1.313, *p =* 0.036), mean corpuscular volume (β ± SE: 0.019 ± 0.008, *p =* 0.014), and transferrin saturation (β ± SE: 0.003 ± 0.001, *p =* 0.006) were independently positively associated with NT_50_. An additional multiple linear regression analysis was conducted with all variables, but excluding anti-S1 and anti-RBD antibodies (Model 2), and found that red blood cells (β ± SE: 6.169 ± 3.063, *p =* 0.046) and mean corpuscular volume (β ± SE: 0.042 ± 0.018, *p =* 0.022) were independently positively associated with NT_50_ for nAbs, while age (β ± SE: −0.009 ± 0.003, *p =* 0.011), K (β ± SE: −0.115 ± 0.057, *p =* 0.046), and cardiothoracic ratio (β ± SE: −2.259 ± 0.719, *p =* 0.002) were independently negatively associated with NT_50_ (Table 2). 

### 3.3. Predictors Associated with Positive Humoral Response in HD Patients

**Figure 2:**
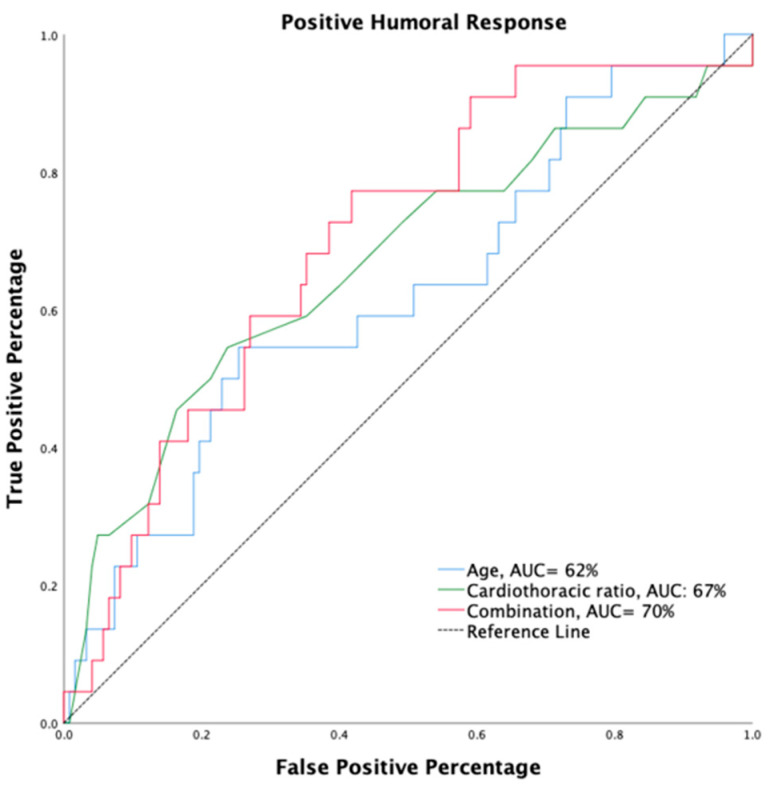
Receiver operating characteristic curve illustrating the performance of age, cardiothoracic ratio, and their combination in predicting the development of NT_50_ values over 35.13 IU/mL after a single dose of ChAdOx1 vaccination.

Binary univariate followed by multivariable logistic regression analyses were applied to evaluate the risk of several dialysis-related parameters associated with high NT_50_. We found that cardiothoracic ratio (OR < 0.001, 95% CI: 0.000–0.556, *p =* 0.037) was independently inversely related to NT_50_ over 35.13 IU/mL, and age (OR: 0.958, 95% CI: 0.917–1.000, *p =* 0.052) also showed a similar tendency (Table 3). Figure 2 shows a receiver operating characteristic curve illustrating the performance of younger age (AUC: 62%), lower cardiothoracic ratio (AUC: 67%), and the combination obtained by multiplying the two (AUC: 70%) in predicting the development of nAb titers over 35.13 IU/mL after a single dose of ChAdOx1 vaccination (Figure 2). The results suggest that there might be an interaction between age and cardiothoracic ratio affecting immunogenicity.

### 3.4. Different Humoral Responses between HD Patients and Healthy Controls

**Figure 3:**
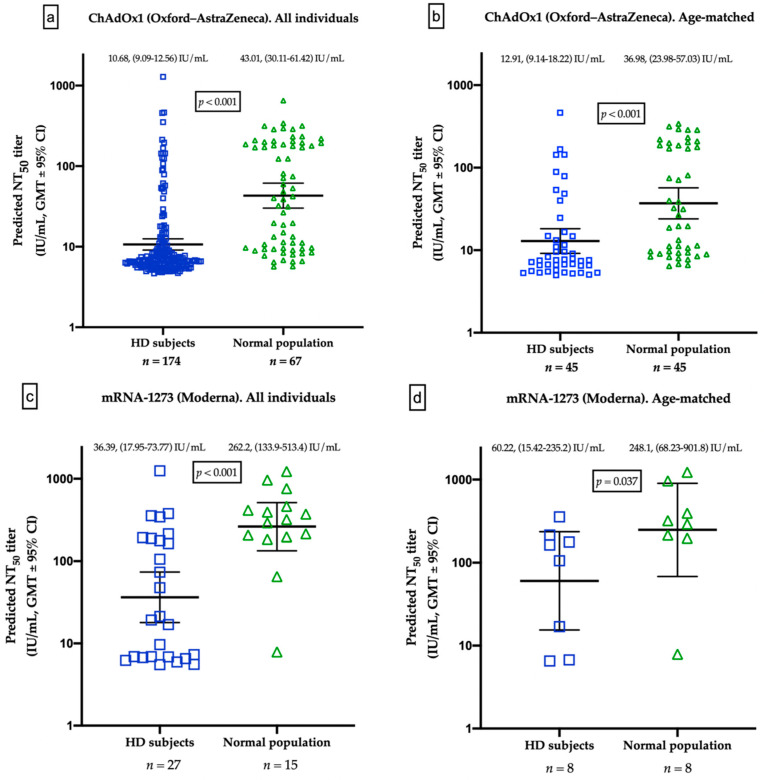
(**a**) Comparison of geometric mean titers (GMTs) for neutralizing antibodies (nAbs) between hemodialysis patients and healthy controls vaccinated with a first dose of ChAdOx1. (**b**) Comparison of GMTs for nAbs from age-matched hemodialysis patients and healthy controls vaccinated with a first dose of ChAdOx1. (**c**) Comparison of geometric mean titers (GMTs) for nAbs from hemodialysis patients and healthy controls vaccinated with a first dose of mRNA-1273. (**d**) Comparison of GMTs for nAbs from age-matched hemodialysis patients and healthy controls vaccinated with a first dose of mRNA-1273.

The healthy controls were all younger than the HD patients (44.86 ± 9.85 vs. 64.97 ± 13.20, *p* < 0.001; and 50.07 ± 19.02 vs. 68.51 ± 10.35, *p =* 0.001, respectively) in both vaccination groups. We obtained GMTs of 10.68 IU/mL and 43.01 IU/mL for nAbs in serum samples from ChAdOx1-vaccinated HD patients and healthy controls (*p* < 0.001), respectively (Table 4, Figure 3a). The GMT values from mRNA-1273-vaccinated HD patients and healthy controls were 36.39 IU/mL and 262.2 IU/mL (*p =* 0.001), respectively (Table 4, Figure 3c). Because of significant differences in age between HD patients and normal controls, a resampling subset of patients with individually matched ages (±2 years) as built. Again, we found that HD patients had lower GMTs for nAbs than healthy controls receiving either ChAdOx1 (12.91 vs. 36.98 IU/mL, *p* < 0.001) or mRNA-1273 (60.22 vs. 248.1 IU/mL, *p* = 0.038) (Figure 3b,d). Specifically, healthy controls receiving mRNA-1273 had a fourfold increase in GMT values compared to HD patients. With regard to positive humoral response rate, HD patients also had a lower rate than that of healthy controls with both types of vaccine (13.2% vs. 52.2%, *p* < 0.001; and 46.2% vs. 93.3%, *p =* 0.004, respectively) (Table 4). This finding confirms the attenuation of humoral response to a single dose of COVID-19 vaccine in uremic patients.

### 3.5. Different Humoral Responses between mRNA and Vector-Based Vaccines

**Figure 4:**
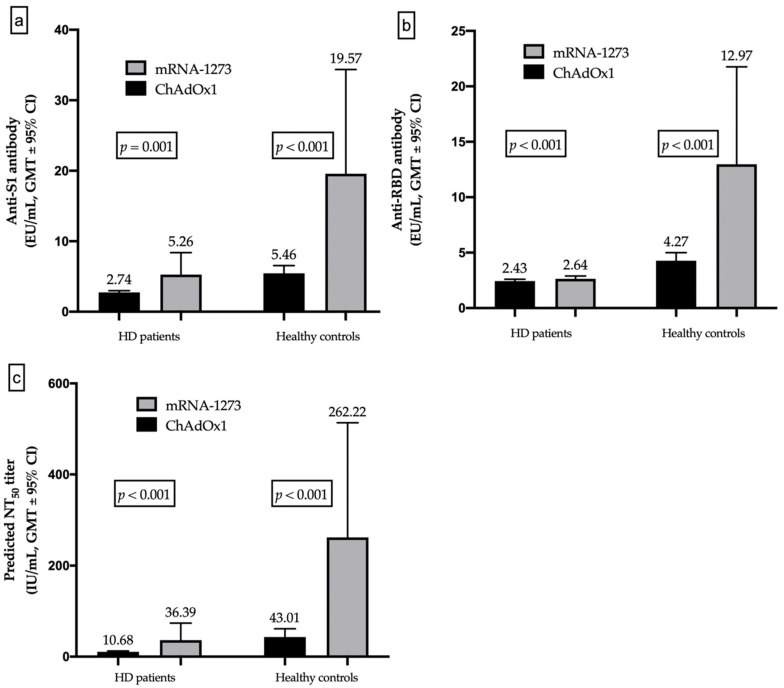
Comparison of humoral responses between ChAdOx1- and mRNA-1273-vaccinated individuals: (**a**) Geometric mean titers (GMTs) of anti-S1 antibodies; (**b**) GMTs of anti-receptor-binding-domain (RBD) antibodies; (**c**) GMTs of predicted SARS-CoV-2 50% neutralization titer (NT_50_).

The adenoviral vector vaccine ChAdOx1 elicited lower humoral response with lower GMTs for anti-S1 and anti-RBD antibodies than the mRNA-1273 vaccine in both HD patients (anti-S1: 2.74 vs. 5.26 EU/mL, *p =* 0.001; anti-RBD: 2.43 vs. 2.64 EU/mL, *p* < 0.001) and healthy controls (anti-S1: 5.46 vs. 19.57 EU/mL, *p* < 0.001; anti-RBD: 4.27 vs. 12.97 EU/mL, *p <* 0.001; Figure 4a,b). Similarly, the GMTs of predicted NT_50_ calculated from GAM regression analysis, using anti-S1 and anti-RBD as predictors, were significantly lower in ChAdOx1-vaccinated compared to mRNA-1273-vaccinated subjects in both the HD (10.68 vs. 36.39, *p* < 0.001) and control groups (43.01 vs. 262.22, *p* < 0.001; Figure 4c). The results suggest that the mRNA-based vaccine mRNA-1273 could elicit more prominent nAb responses than the adenoviral vector vaccine ChAdOx1 in both HD patients and healthy controls.

## 4. Discussion

In this prospective observational single-center study, we demonstrated that HD patients had decreased humoral responses to COVID-19 vaccination compared to healthy controls. Older age and high cardiothoracic ratio were independently associated with lower NT_50_ of nAbs in HD patients receiving a single dose of ChAdOx1 vaccination. The relationship remained significant after adjusting for albumin, gender, and diabetes. However, the GMTs for nAbs were more prominent after a single dose of mRNA-1273 vaccination than after ChAdOx1 vaccination, in both HD patients and healthy controls. 

A single dose of ChAdOx1 vaccine achieves a 76% efficacy for symptomatic COVID-19 after 21 days post-vaccination, and an extension of the dose interval to 12 weeks might provide advantages over an interval of less than 6 weeks [27]. We obtained nAb titers from HD patients on day 56, and from healthy controls on a median of day 30 post-vaccination. However, anti-spike-protein antibodies elicited by ChAdOx1 peaked on day 28, and stayed elevated on day 56 as well as the comparable neutralization test, while Voysey et al. showed only a mild decrease of anti-spike-protein antibodies on day 56 compared with day 28 [27,28]. This phenomenon suggests that the different time of blood sampling between the two groups had a limited influence on the results of our study. In the early stage of the outbreak in mid-May 2021, Taiwan was facing serious difficulties in obtaining vaccines; the initial acquired vaccines were mainly ChAdOx1. Perhaps the results of the above-mentioned research could give Taiwan’s health authorities more confidence to generally promote first-dose vaccination and postpone the second dose when supplies are scarce.

Many studies have evaluated mRNA vaccines in dialysis patients, but only a few studies are available on vector-based vaccines [15,16,29,30,31,32]. The findings of our study are comparable to those of several other studies, showing that the mRNA-1273 vaccine can elicit higher nAb titers than the ChAdOx1 vaccine in both HD patients and healthy controls. Garcia et al. conducted a study assessing the humoral response in dialysis patients by estimating total anti-RBD response, including IgG and IgM titers, and found that one-third of patients (*n =* 367) inoculated with the adenovirus Ad26.COV 2.S (Johnson & Johnson, attenuated adenovirus) vaccine failed to seroconvert. In that cohort, 36% of vaccinated subjects had no detectable or diminished IgG response even 28–60 days post-vaccination [15]. Candice et al. showed that ChAdOx1 vaccination provided inferior humoral response compared to the mRNA vaccine BNT162b2 (BioNTech/Pfizer) in an HD cohort assessed by titers of anti-S antibodies (*n =* 272) [29]. Although the shortage of vaccines is a global challenge, the findings of this and other studies highlight the importance of application of effective vaccines in the most vulnerable populations, such as HD patients. 

Most studies assessed humoral responses by estimating anti-S1 or anti-RBD IgG or IgM as a surrogate for nAbs; however, these antibodies might indicate past or recent exposure to COVID-19, rather than neutralizing ability. Investigations concerning viral neutralization tests in HD cohorts remain scarce. Only a UK study applied live virus microneutralization in seronegative HD patients, comparing ChAdOx1 (*n =* 53) and BNT162b2 (*n =* 55) response, and found that the mRNA vaccine provokes comparable nAb titers in HD patients and healthy controls, while ChAdOx1 induces suboptimal nAb titers against all variants of concern (VOCs), including the delta variant that is dominant worldwide [16]. Our study used ELISA procedures to detect concentrations of both anti-S1 and anti-RBD antibodies, including IgG, IgM, and IgA, and the results were computed by a spline-based two-variable GAM to approximate real NT_50_ titers for nAbs; the correlation between predicted and actual virus neutralization titers was as high as 0.917, according to the manufacturer. Real NT_50_ (IU/mL) from a biosafety level 3 laboratory was used as a standard to assess whether these widely used commercial serological assays reflect the neutralization titers based on the detection of antibodies against S1 and/or RBD. The highest correlation was observed between the titers obtained by the MeDiPro and the live SARS-CoV-2 neutralization test assays (r = 0.9111), which were superior to widely used commercial assays such as the Roche and Abbott RBD antibody titers, with fair correlation coefficients of 0.7294 and 0.8466, respectively [33]. Accordingly, this method might serve as a surrogate for the rapid quantification of nAb titers, replacing the conventional laboratories and time-consuming virus neutralization tests. Therefore, it represents a safer, quicker, and more efficient way to quantify nAbs than other tests. To our knowledge, our study is the first attempt to explore using the predicted NT_50_ for quantifying the nAb response in an extensive cohort of HD patients (*n =* 200) vaccinated with ChAdOx1.

The definition of positive humoral response rate used for our laboratory method is the predicted NT_50_ ≥ 35.13 IU/mL, which is more stringent than previous studies using occurrence of IgG or IgM as indicators of seroconversion. This may in part underestimate the response rate of our HD patients compared to the levels of previous studies. Moreover, the response rate was indeed lower in HD patients than in healthy controls, especially in the ChAdOx1 group (13.2%). Uremic patients have defects in innate and adaptive immunity [34]; in the uremic milieu, antigen-presenting cell dysfunction and memory T-cell apoptosis may reduce antibody production by B cells. In addition, signals of B-cell differentiation are also damaged [35,36,37]. The impairment of antigen-presenting capability further makes the immune systems of uremic patients struggle to recognize pathogens and to produce a series of downstream adaptive immunity [38,39]. Though humoral immunity cannot be representative of overall immunity, and the ChAdOx1 vaccine was reported to elicit more prominent cellular immunity than mRNA vaccines, the lack of an nAb response was a strong predictor of death, adjusted for age and gender [40,41,42]. This phenomenon suggests that immunocompromised subjects—including those with uremia, solid organ transplants, or malignancy—might receive a heterologous vaccination strategy with an mRNA vaccine as a second dose to boost serum nAb quantity, if primed with ChAdOx1 as the first dose.

Diabetes, albumin, C-reactive protein, and Kt/V were important indicators of clinical outcomes in HD patients; however, these parameters were not correlated with levels of nAb production in our study. Our findings were similar to those of a study on Mexico’s healthcare workers, showing that gender and comorbidities (such as diabetes, obesity, and hypertension) are not associated with low titers of nAbs [43,44]. A relatively homogeneous status regarding nutrition, inflammation, and administered dialysis dosage may all contribute to this phenomenon.

Cardiothoracic ratio is one of the monitoring indicators of dialysis quality, and is usually used to assess the fluid status of dialysis patients, and as an important reference for dry weight adjustment. Cardiothoracic ratio has been found to be positively associated with C-reactive protein and inversely associated with albumin, and can predict 2-year all-cause mortality and infection-caused mortality in HD populations [45,46,47]. High cardiothoracic ratio is also reported to be a significant and independent predictor of cardiovascular events in patients undergoing HD [48,49,50]. Cardiovascular disease has been viewed as a risk factor for severe COVID-19, and is strongly associated with in-hospital death [51,52]. High cardiothoracic ratio has been linked to chronic inflammation and malnutrition in HD patients, and the status of chronic inflammation might hinder the production of nAbs [53]. This phenomenon possibly illustrates the connection between high cardiothoracic ratio and low nAb titers found in our study. 

This study has some limitations. First, a single dose of COVID-19 vaccination could not precisely predict the final results after full vaccination—it simply reflects the humoral response during that period when Taiwan faced a shortage of vaccines, and even the prioritized, vulnerable patients only received a single dose. Second, we did not measure nAbs before vaccination. There was almost no domestic transmission before May 2021 in Taiwan, and there were no confirmed cases in our HD center, in combination with weekly rapid antigen testing on our HD patients in case of asymptomatic infection; hence, the patients were supposed to be negative for nAbs. Third, we used a relatively small sample, and we only performed further analyses in the ChAdOx1 group to explore potential factors associated with responses to vaccination. The mRNA-1273 group and control group enrolled fewer people, and the subjects in the HD cohort and healthy controls were highly heterogeneous in terms of age and sex. Hence, we built a resampling subset of patients individually matched for age, which yielded similar results. Nevertheless, to our knowledge, we used the largest number of HD patients receiving ChAdOx1 enrolled for NT_50_ analyses compared with previous studies. Fourth, we did not conduct baseline laboratory testing of healthy controls, because they were generally healthy, and we simply collected blood samples for measurement of nAbs. Finally, NT_50_ titers were not predicted through real neutralization testing in our study; however, the assay we used has been proven to have better correlation with real neutralization tests than other commercial serological tests [33].

## 5. Conclusions

This prospective, observational, single-center study established the associations of high cardiothoracic ratio and old age with reduced nAb titers in an HD cohort vaccinated with a single dose of ChAdOx1. The GMTs for nAbs were lower in HD patients vaccinated with ChAdOx1 or mRNA-1273. mRNA-1273 could elicit higher nAbs and positive humoral response rate in both HD patients and healthy controls.

We also noticed that a single dose of ChAdOx1 only provided 13.2% of HD patients with sufficient nAbs, while mRNA-1273 provided close to half of HD patients with a positive humoral response. To enhance dialysis quality and avoid severe COVID-19, maintaining adequate cardiothoracic ratio by close monitoring of fluid status is worthy of our scrutiny, and we should also consider offering an mRNA vaccine as a second dose to boost nAb quantity if patients were vaccinated with ChAdOx1 as a first dose.

## Figures and Tables

**Table 1:** Baseline characteristics of patients receiving a single dose of ChAdOx1 vaccine, stratified by NT_50_ titer.

	NT_50_ < 12.31 IU/mL (*n* = 135)	12.31 ≤ NT_50_ Titer < 35.13 (*n =* 16)	NT_50_ Titer ≥ 35.13 (*n* = 23)	*p*-Value
Age, year	65.67 ± 12.76	61.69 ± 13.70	60.70 ± 14.63	0.094 ^&^
Male, *n* (%)	72 (53.3)	8 (50)	14 (60.9)	0.754
Comorbidities, *n* (%)				
Diabetes	74 (54.8)	9 (56.3)	13 (56.5)	0.984
Dyslipidemia	49 (36.3)	8 (50)	10 (43.5)	0.494
Liver cirrhosis	4 (3)	0 (0)	0 (0)	0.551
Cardiovascular disease	49 (36.3)	7 (43.8)	8 (34.8)	0.824
Baseline medications, *n* (%)				
Immunosuppressants	8 (5.9)	1 (6.3)	0 (0)	0.485
RAAS blockade	60 (44.4)	4 (25)	11(47.8)	0.294
β-blockers	55 (40.7)	6 (37.5)	12 (52.2)	0.549
Statins	45 (33.3)	7 (43.8)	10 (43.5)	0.500
Anti-S1 antibody (EU/mL)	2.11 (1.96–2.41)	3.49 (3.14–3.86)	5.99 (5.18–9.61)	<0.001 ^$^
Anti-RBD antibody (EU/mL)	2.07 (1.86–2.26)	3.16 (3.04–3.55)	3.79 (3.22–5.37)	<0.001 ^$^
Hemoglobin (g/dL)	10.04 ± 1.14	10.23 ± 1.55	10.20 ± 1.19	0.561 ^&^
WBC (1000/μL)	5.90 (4.70–7.50)	7.10 (5.80-7.85)	6.30 (5.30–7.80)	0.183 ^$^
Platelet (1000/μL)	187.65 ± 65.41	240.56 ± 86.20	189.52 ± 76.62	0.905 ^&^
Albumin (g/dL)	4.025 ± 0.38	3.96 ± 0.32	4.09 ± 0.32	0.465 ^&^
Cholesterol (mg/dL)	154.85 ± 37.23	160.44 ± 32.32	147.91 ± 35.72	0.556 ^&^
Triglyceride (mg/dL)	114.00 (78.00–173.50)	152.50 (111.00–304.25)	128.00 (77.00–164.00)	0.160 ^$^
AST (U/L)	16.0 (13.0–20.5)	18.50 (14.00–25.25)	17 (14–21)	0.380 ^$^
ALT (U/L)	13 (10–19)	16.50 (10.25–21.50)	17 (13–21)	0.043 * ^$^
Alk-P (U/L)	92 (71–146)	114.00 (80.00–235.25)	79 (65–116)	0.136 ^$^
Total bilirubin (mg/dL)	0.40 (0.30–0.40)	0.35 (0.20–0.40)	0.40 (0.30–0.40)	0.749 ^$^
Bun (mg/dL)	69.97 ± 22.94	68.73 ± 19.43	67.99 ± 12.97	0.686 ^&^
Creatinine (mg/dL)	9.65 ± 2.59	9.22 ± 3.14	10.06 ± 1.88	0.475 ^&^
Uric acid (mg/dL)	6.28 ± 1.95	6.21 ± 1.83	6.18 ± 1.84	0.810 ^&^
Na (meq/L)	138.14 ± 3.08	137.5 ± 3.86	138.43 ± 2.73	0.676 ^&^
K (meq/L)	4.76 ± 0.80	4.77 ± 0.78	4.57 ± 0.71	0.291 ^&^
Ca (mg/dL)	9.35 ± 0.85	9.39 ± 0.78	9.67 ± 0.62	0.081 ^&^
P (mg/dL)	5.26 ± 1.59	5.41 ± 1.95	5.36 ± 1.45	0.772 ^&^
C-reactive protein (mg/L)	3.60 (1.30–9.20)	6.30 (2.70–9.90)	4.45 (1.63–9.30)	0.457 ^$^
Urea reduction rate	76.00 (70.50–79.50)	75.00 (68.25–80.00)	76 (72–79)	0.695 ^$^
Kt/V (Daugirdes)	0.65 ± 0.34	1.61 ± 0.29	1.60 ± 0.35	0.562 ^&^
nPCR (g/kg/day)	1.10 ± 0.58	1.00 ± 0.28	1.04 ± 0.25	0.624 ^&^
TACurea	41.69 ± 14.14	42.00 ± 12.18	39.72 ± 9.97	0.519 ^&^
Iron (μg/dL)	65.00 (50.50–89.00)	55 (46–81)	78 (54–103)	0.280 ^$^
Ferritin (ng/mL)	439.00 (234.00–709.50)	338.50 (171.50–516.00)	524 (120–600)	0.256 ^$^
TSAT (%)	34.02 ± 13.68	29.77 ± 8.66	37.82 ± 19.72	0.240 ^&^
Cardiothoracic ratio	0.53 ± 0.05	0.48 ± 0.06	0.48 ± 0.07	0.006 *
Ca × P product	49.38 ± 16.95	50.66 ± 18.43	52.21 ± 16.01	0.461

Notes: Data are presented as mean ± standard deviation or median (interquartile range). Abbreviations—RAAS: renin–angiotensin–aldosterone system; WBC: white blood cell count; AST: aspartate transaminase; ALT: alanine transaminase; Alk-P: alkaline phosphatase; Bun: blood urea nitrogen; Kt/V: a mathematical formula representing a dose of dialysis; nPCR: normalized protein catabolic rate; NT_50:_ predicted SARS-CoV-2 50% neutralization titer, expressed in international units; TACurea: time-averaged urea concentration; TSAT: transferrin saturation. *: statistically significant. ^$^: comparison among HD patients with different humoral responses by nonparametric independent Kruskal–Wallis test. ^&^: Comparison among HD patients with different humoral responses by one-way ANOVA.

**Table 2:** β-coefficients between predicted NT_50_ and independent variables.

	Simple Linear Regression		Multiple Regression Analysis, Model 1		Multiple Regression Analysis, Model 2	
	β ± SE	*p*	β ± SE	*p*	β ± SE	*p*
Age	−0.005 ± 0.003	0.075	−0.003 ± 0.001	0.046 *	−0.009 ± 0.003	0.011 *
Anti-S1 antibody ^$^	1.841 ± 0.065	<0.001 *	1.490 ± 0.128	<0.001 *	-	-
Anti-RBD antibody ^$^	2.250 ± 0.0106	<0.001 *	0.589 ± 0.165	0.001 *	-	-
Hemoglobin	0.004 ± 0.032	0.890	−0.136 ± 0.056	0.017 *	−0.251 ± 0.130	0.054
RBC ^$^	−0.225 ± 0.623	0.718	2.787 ± 1.313	0.036 *	6.169 ± 3.063	0.046 *
MCV	0.004 ± 0.005	0.478	0.019 ± 0.008	0.014 *	0.042 ± 0.018	0.022 *
WBC ^$^	0.375 ± 0.246	0.129	-	-	-	-
Platelet	0.001 ± 0.001	0.254	-	-	-	-
Albumin	0.061 ± 0.104	0.560	-	-	-	-
AST ^$^	0.011 ± 0.222	0.959	-	-	-	-
ALT ^$^	0.308 ± 0.167	0.066	-	-	0.401 ± 0.191	0.038 *
Alk-P ^$^	−0.120 ± 0.137	0.380	-	-	-	-
Bilirubin ^$^	−0.047 ± 0.252	0.853	-	-	-	-
Cholesterol	−0.001 ± 0.001	0.564	-	-	-	-
Triglyceride ^$^	0.034 ± 0.151	0.822	-	-	-	-
Creatinine	0.007 ± 0.015	0.628	0.016 ± 0.009	0.073	-	-
Uric acid	0.004 ± 0.020	0.858	−0.021 ± 0.010	0.038 *	-	-
Na	0.006 ± 0.012	0.632	-	-	-	-
K	−0.049 ± 0.048	0.312	-	-	−0.115 ± 0.057	0.046 *
Ca	0.083 ± 0.046	0.074	-	-	-	-
P	0.024 ± 0.024	0.320	-	-	-	-
C-reactive protein ^$^	0.072 ± 0.064	0.262	-	-	-	-
URR ^$^	0.038 ± 0.240	0.875	−0.655 ± 0.354	0.067	-	-
Kt/V	−0.048 ± 0.117	0.680	-	-	-	-
nPCR	−0.043 ± 0.074	0.559	-	-	-	-
TACurea	−0.001 ± 0.003	0.860	-	-	-	-
Ferritin ^$^	−0.062 ± 0.095	0.516	−0.119 ± 0.048	0.016 *	-	-
Iron ^$^	0.245 ± 0.216	0.258	-	-	-	-
TSAT	0.002 ± 0.003	0.465	0.003 ± 0.001	0.006 *	-	-
Intact-PTH	−0.000 ± 0.000	0.503	-	-	-	-
Cardiothoracic ratio	−2.026 ± 0.729	0.006 *	−0.555 ± 0.305	0.071	−2.259 ± 0.719	0.002 *
Ca × P product	0.003 ± 0.002	0.158	−0.003 ± 0.001	0.006 *	-	-

A backward stepwise selection method was applied for multivariate analysis. Abbreviations—WBC: white blood cell count; AST: aspartate transaminase; ALT: alanine transaminase; Alk-P: alkaline phosphatase; Bun: blood urea nitrogen; Kt/V: a mathematical formula representing a dose of dialysis; NT_50_: SARS-CoV-2 50% neutralization titer, expressed in international units; nPCR: normalized protein catabolic rate; TACurea: time-averaged urea concentration; TSAT: transferrin saturation. *: Statistically significant; ^$^: Log_10_ transformed.

**Table 3:** Binary logistic regression of factors associated with positive humoral response (NT_50_ > 35.13 IU/mL) to a single dose of ChAdOx1 vaccine.

	Univariate			Multivariable		
	OR	95% CI	*p*-Value	OR	95% CI	*p*-Value
Male	0.724	0.296–1.775	0.481	0.809	0.214–3.056	0.755
Age	0.975	0.944–1.007	0.125	0.958	0.917–1.000	0.052
Diabetes	0.939	0.388–2.274	0.889	0.849	0.285–2.528	0.849
Liver cirrhosis	2.54 × 10^8^	0	0.999	1.09 × 10^7^	0	1.000
Cardiovascular disease	1.105	0.441–2.772	0.831	0.915	0.269–3.116	
Immunosuppressants	0	0	0.999	2.37 × 10^−9^	0	0.999
RAAS blockade	1.246	0.517–3.003	0.624	1.132	0.348–3.677	0.837
β-blockers	1.610	0.667–3.882	0.289	0.612	0.199–1.885	0.392
Hemoglobin	1.102	0.760–1.599	0.608	0.773	0.161–3.721	0.748
RBC	0.993	0.440–2.239	0.987	1.934	0.015–252.653	0.791
MCV	1.020	0.955–1.090	0.560	1.078	0.853–1.364	0.528
Albumin	1.700	0.482–5.999	0.410	0.852	0.119–6.097	0.873
ALT	1.009	0.981–1.039	0.526	1.007	0.969–1.046	0.720
K	0.727	0.407–1.301	0.283	0.625	0.273–1.432	0.267
C-reactive protein	0.945	0.499–1.789	0.861	1.008	0.972–1.045	0.670
Kt/V	0.700	0.190–2.570	0.591	0.977	0.131–7.294	0.982
nPCR	0.776	0.226–2.663	0.687	0.556	0.053–5.784	0.623
TSAT	1.019	0.991–1.047	0.189	1.015	0.982–1.049	0.390
Cardiaothoracic ratio	0.005	0.000–0.307	0.012	<0.0001	0.000–0.556	0.037 *
Ca × P product	1.009	0.984–1.035	0.477	1.019	0.981–1.059	0.325

Abbreviations—RAAS: renin–angiotensin–aldosterone system; RBC: red blood cell count; MCV: mean corpuscular volume; ALT: alanine transaminase; Kt/V: a mathematical formula representing a dose of dialysis; nPCR: normalized protein catabolic rate; NT_50_: SARS-CoV-2 50% neutralization titer, expressed in international units; TSAT: transferrin saturation; *: Statistically significant.

**Table 4:** Different humoral responses to two types of COVID-19 vaccine.

	ChAdOx1n (Oxford–AstraZeneca)			mRNA-1273 (Moderna)		
	HD Group (*n =* 174)	Control Group (*n =* 67)	*p*-Value	HD Group (*n =* 26)	Control Group (*n =* 15)	*p*-Value
Age (years)	64.97 ± 13.20	44.86 ± 9.85	<0.001 *	68.51 ± 10.35	50.07 ± 19.02	0.001 *
Male, *n* (%)	94 (54)	22 (32.8)	0.003 *	7 (26.9)	13 (86.7)	<0.001 *
Predicted NT_50_ titer (median, IQR) (IU/mL)	6.85 (5.89–10.68)	40.01 (9.58–186.71)	<0.001 *	20.27 (6.83–189.66)	319.44 (196.54–461.48)	0.001 *
GMTs (95% CI) (IU/mL)	10.68 (9.10–12.54)	43.01 (30.11–61.42)		36.39 (17.95–73.77)	262.2 (133.9–513.4)	
Humoral response, *n* (%)	23 (13.2)	35 (52.2)	<0.001 *	12 (46.2)	14 (93.3)	0.004 *

Notes: Data are presented as mean ± standard deviation or median (interquartile range). Abbreviations—NT_50_: SARS-CoV-2 50% neutralization titer, expressed in international units; GMTs: geometric mean titers; Humoral responses were defined as having predicted NT_50_ > 35.13 IU/mL. *: Statistically significant.

## Data Availability

Not applicable.

## Supplementary Materials

The following supporting information can be downloaded at https://www.mdpi.com/article/10.3390/jpm12010068/s1: Table S1: Comparisons of demographic and clinical characteristics between enrolled and non-enrolled patients on hemodialysis; Table S2: Comparisons of demographic and clinical characteristics between ChAdOx1- (Oxford–AstraZeneca) and mRNA-1273 (Moderna)-vaccinated patients on hemodialysis; Figure S1: Correlation between the age and predicted SARS-CoV-2 50% neutralization titer (NT_50_) in the ChAdOx1 group (Panel a) and mRNA-1273 group (Panel b).
