# Supplementary material for: Neutralization Assessments Reveal High Cardiothoracic Ratio and Old Age as Independent Predictors of Low Neutralizing Antibody Titers in Hemodialysis Patients Receiving a Single Dose of COVID-19 Vaccine
Source: J Pers Med. 2022 Jan 7;12(1):68. doi: 10.3390/jpm12010068 (PMC8781271; doi:10.3390/jpm12010068)
Supplement: Supplementary file 1 [file jpm-12-00068-s001.zip › jpm-1537221-supplementary.pdf]

Table S1. Demographics and clinical characteristics comparisons between enrolled and non-enrolled patients on hemodialysis

	**Enrolled (*n* = 200)**	**Non-enrolled (*n* = 298)**	***p* value**
Age, year	65.08 ± 12.87	66.44 ± 13.55	0.264
Male, n (%)	101 (50.5)	163 (54.7)	0.357
Anti-S1 Abs (EU/mL)	2.33 (2.01-3.45)	N/A	N/A
Anti-RBD Abs (EU/mL)	2.15 (1.94-2.92)	N/A	N/A
Hemoglobin (g/dL)	10.06 ± 1.16	10.06 ± 1.31	0.993
WBC (1000/μL)	6.45 ± 2.74	6.73 ± 2.96	0.290
Platelet (1000/μL)	190.93 ± 67.60	190.01 ± 69.23	0.884
Albumin (g/dL)	4.04 ± 0.37	3.97 ± 0.55	0.149
Cholesterol (mg/dL)	154.83 ± 35.78	152.86 ± 36.66	0.562
Triglyceride (mg/dL)	115 (78.75-175.25)	122 (81-184)	0.540$
AST (U/L)	17 (13-21)	17 (13-21)	0.996$
ALT (U/L)	14 (10-19)	15 (11-20)	0.036*$
Alk-P (U/L)	92 (72.25-137.75)	92 (71-136)	0.732$
Total bilirubin (mg/dL)	0.4 (0.3-0.4)	0.3 (0.3-0.4)	0.398$
Bun (mg/dL)	69.93 ± 21.13	71.12 ± 22.03	0.548
Creatinine (mg/dL)	9.64 ± 2.50	9.22 ± 2.80	0.086
Uric acid (mg/dL)	6.22 ± 1.92	6.58 ± 1.73	0.032*
Na (meq/L)	138.11 ± 3.15	138.07 ± 3.25	0.914
K (meq/L)	4.77 ± 0.79	4.65 ± 0.85	0.140
Ca (mg/dL)	9.40 ± 0.81	9.28 ± 0.94	0.160
P (mg/dL)	5.34 ± 1.57	5.21 ± 1.54	0.383
C-reactive protein(mg/L)	4.30 (1.40-9.42)	3.35 (1.20-9.20)	0.482$
Urea reduction rate	76 (71-80)	76 (71-80)	0.961$
Kt/V (Daugirdes)	1.65 ± 0.33	1.68 ± 0.32	0.421
nPCR (g/kg/day)	1.09 ± 0.49	1.07 ± 0.29	0.660
TACurea	41.48 ± 13.16	42.16 ± 13.46	0.586
Iron (μg/dL)	68 (51-90)	65 (50.00-82.25)	0.167$
Ferritin (ng/mL)	403.50 (223.00-654.75)	437.00(173.00-675.25)	0.860$
TSAT (%)	34.02 ± 13.89	32.23 ± 14.91	0.185
Cardiothoracic ratio	0.52 ± 0.06	0.52 ± 0.07	0.563
Ca × P product	50.32 ± 16.42	48.38 ± 15.09	0.176

Notes: Data are presented as mean ± standard deviation and median (interquartile range). Abbreviations: Abs, antibodies; WBC, white blood cell count; AST, aspartate transaminase; ALT, alanine transaminase; Alk-P, alkaline phosphatase; Bun, blood urea nitrogen; Kt/V, A mathematical formula representing a dose of dialysis; nPCR, normalized protein catabolic rate; N/A, not applicable; TACurea, time average urea concentration; TSAT, transferrin saturation. *: statistically significant; $: Nonparametric, independent Sample Mann-Whitney U test.

Table S2. Demographics and clinical characteristics comparisons between ChAdOx1 (Oxford–AstraZeneca) and mRNA-1273 (Moderna) vaccinated patients on hemodialysis

	**ChAdOx1(*n* = 174)**	**mRNA-1273 (*n* = 26)**	***p* value**
Age, year	64.97 ± 13.20	68.51 ± 10.35	0.192
Male, n (%)	94 (54)	7 (26.9)	0.01*
Diabetes, n (%)	96 (55.2)	15 (57.7)	0.809
Liver cirrhosis, n (%)	4 (2.3)	4 (15.4)	0.002*
Dyslipidemia, n (%)	67 (38.5)	12 (46.2)	0.457
Cardiovascular disease, n (%)	64 (36.8)	3 (11.5)	0.011*
Immunosuppressant, n (%)	9 (5.2)	0 (0)	0.609
RAAS blockade, n (%)	75 (43.1)	12 (46.2)	0.770
β-blocker, n (%)	73 (42.0)	7 (26.9)	0.144
Statin, n (%)	62 (35.6)	11 (42.3)	0.510
Anti-S1 antibody (EU/mL)	2.28 (1.99-3.13)	3.89 (2.24-9.07)	0.001*$
Anti-RBD antibody (EU/mL)	2.14 (1.92-2.64)	3.16 (2.14-6.84)	<0.001*$
Hemoglobin (g/dL)	10.08 ± 1.18	9.95 ± 1.01	0.611
WBC (1000/μL)	6.05 (4.90-7.63)	5.55 (4.63-6.80)	0.259$
Platelet (1000/μL)	192.76 ± 70.27	178.65 ± 44.93	0.322
Albumin (g/dL)	4.027 ± 0.37	4.095 ± 0.40	0.388
Cholesterol (mg/dL)	154.44 ± 36.53	157.38 ± 30.84	0.697
Triglyceride (mg/dL)	116.50 (79.25-177.50)	108.00 (71.75-149.50)	0.532$
AST (U/L)	17 (13-21)	16.5 (14.0-21.5)	0.812$
ALT (U/L)	14 (10-19)	13.00 (10.75-17.25)	0.717$
Alk-P (U/L)	92.0(71.0-138.5)	97.50 (81.75-140.25)	0.316$
Total bilirubin (mg/dL)	0.4 (0.3-0.4)	0.40 (0.30-0.43)	0.554$
Bun (mg/dL)	69.59 ± 21.49	71.16 ± 18.71	0.565
Creatinine (mg/dL)	9.66 ± 2.56	9.49 ± 2.10	0.748
Uric acid (mg/dL)	6.26 ± 1.92	5.92 ± 1.96	0.398
Na (meq/L)	138.12 ± 3.11	138.04 ± 3.53	0.902
K (meq/L)	4.74 ± 1.79	4.95 ± 0.77	0.208
Ca (mg/dL)	9.40 ± 0.83	9.40 ± 0.76	0.997
P (mg/dL)	5.28 ± 1.60	5.68 ± 1.34	0.231
C-reactive protein	4.35 (1.40-10.03)	3.50 (1.13-6.73)	0.282$
Urea reduction rate	76 (71-79)	77.50 (73.00-81.25)	0.055$
Kt/V (Daugirdes)	1.64 ± 0.34	1.76 ± 0.26	0.079
nPCR (g/kg/day)	1.09 ± 0.52	1.09 ± 0.23	0.991
TACurea	41.46 ± 13.44	41.63 ± 11.37	0.952
Iron (μg/dL)	65.00 (50.25-89.75)	74.00 (58.75-95.25)	0.238$
Ferritin (ng/mL)	417.00 (221.75-681.25)	364.50 (221.75-584.00)	0.252$
TSAT (%)	34.13 ± 14.30	33.31 ± 10.93	0.780
Cardiothoracic ratio	0.52 ± 0.07	0.52 ± 0.07	0.928
Ca × P product	49.87 ± 16.89	53.32 ± 12.70	0.319

Notes: Data are presented as mean ± standard deviation or median (interquartile range). Abbreviations: WBC, white blood cell count; AST, aspartate transaminase; ALT, alanine transaminase; Alk-P, alkaline phosphatase; Bun, blood urea nitrogen; Kt/V, A mathematical formula representing a dose of dialysis;

nPCR, normalized protein catabolic rate; TACurea, time average urea concentration; TSAT, transferrin saturation. *: statistically significant; $: Nonparametric, independent-Sample Mann-Whitney U test.

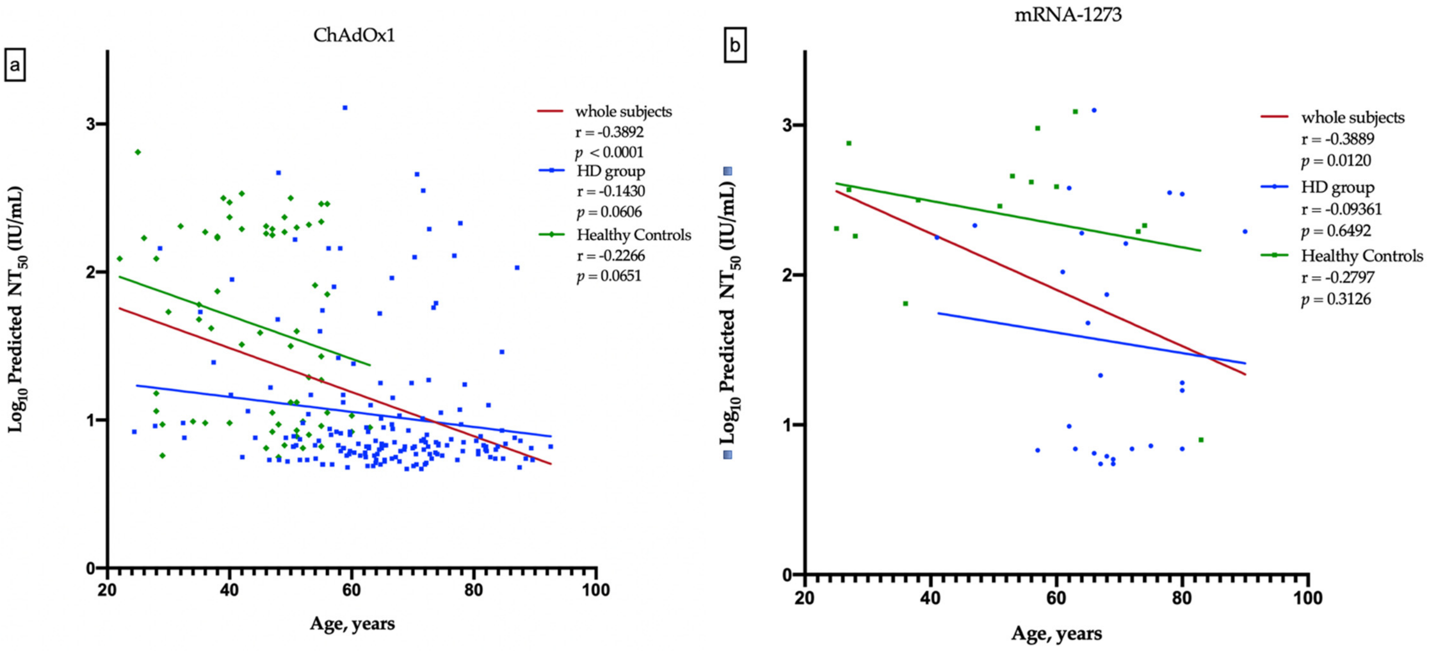

Figure S1. Correlation between the age and predicted SARS-CoV-2 50% neutralization titer (NT_{50}) in ChAdOx1 group (Panel a) and mRNA-1273 group (Panel b).
